# Exploring Discussions About Virtual Reality on Twitter to Inform Brain Injury Rehabilitation: Content and Network Analysis

**DOI:** 10.2196/45168

**Published:** 2024-01-19

**Authors:** Sophie Brassel, Melissa Brunner, Andrew Campbell, Emma Power, Leanne Togher

**Affiliations:** 1 Sydney School of Health Sciences Faculty of Medicine and Health The University of Sydney Sydney Australia; 2 School of Medical Sciences Faculty of Medicine and Health The University of Sydney Sydney Australia; 3 Graduate School of Health University of Technology Sydney Sydney Australia; 4 Western Sydney Local Health District Sydney Australia

**Keywords:** virtual reality, Twitter, brain injury, rehabilitation, cognitive communication, social networks, social media, brain injury rehabilitation, engagement, development, clinical practice, injury, users

## Abstract

**Background:**

Virtual reality (VR) use in brain injury rehabilitation is emerging. Recommendations for VR development in this field encourage end user engagement to determine the benefits and challenges of VR use; however, existing literature on this topic is limited. Data from social networking sites such as Twitter may further inform development and clinical practice related to the use of VR in brain injury rehabilitation.

**Objective:**

This study collected and analyzed VR-related tweets to (1) explore the VR tweeting community to determine topics of conversation and network connections, (2) understand user opinions and experiences of VR, and (3) identify tweets related to VR use in health care and brain injury rehabilitation.

**Methods:**

Publicly available tweets containing the hashtags #virtualreality and #VR were collected up to twice weekly during a 6-week period from July 2020 to August 2020 using NCapture (QSR International). The included tweets were analyzed using mixed methods. All tweets were coded using inductive content analysis. Relevant tweets (ie, coded as “VR in health care” or “talking about VR”) were further analyzed using Dann’s content coding. The biographies of users who sent relevant tweets were examined descriptively. Tweet data networks were visualized using Gephi computational analysis.

**Results:**

A total of 260,715 tweets were collected, and 70,051 (26.87%) were analyzed following eligibility screening. The sample comprised 33.68% (23,596/70,051) original tweets and 66.32% (46,455/70,051) retweets. Content analysis generated 10 main categories of original tweets related to VR (ie, advertising and promotion, VR content, talking about VR, VR news, general technology, VR industry, VR live streams, VR in health care, VR events, and VR community). Approximately 4.48% (1056/23,596) of original tweets were related to VR use in health care, whereas 0.19% (45/23,596) referred to VR in brain injury rehabilitation. In total, 14.86% (3506/23,596) of original tweets featured commentary on user opinions and experiences of VR applications, equipment, and software. The VR tweeting community comprised a large network of 26,001 unique Twitter users. Users that posted tweets related to “VR in health care” (2124/26,001, 8.17%) did not form an interconnected VR network, whereas many users “talking about VR” (3752/26,001, 14.43%) were connected within a central network.

**Conclusions:**

This study provides valuable data on community-based experiences and opinions related to VR. Tweets showcased various VR applications, including in health care, and identified important user-based considerations that can be used to inform VR use in brain injury rehabilitation (eg, technical design, accessibility, and VR sickness). Limited discussions and small user networks related to VR in brain injury rehabilitation reflect the paucity of literature on this topic and the potential underuse of this technology. These findings emphasize that further research is required to understand the specific needs and perspectives of people with brain injuries and clinicians regarding VR use in rehabilitation.

## Introduction

### Background

Acquired brain injuries are a global public health concern [1] and refer to brain injuries sustained after birth, including stroke and traumatic brain injury (TBI) [2,3]. Brain injuries result in substantial socioeconomic burdens [4] and considerable impacts on quality of life [5]. In addition to physical, cognitive, and communication impairments, people with brain injuries can experience challenges in vocational [6,7], social, and community participation [5].

Resources for brain injury rehabilitation may be limited and increasingly challenged owing to the rising incidence and associated long-term care requirements [1,8]. The use of digital health and related technologies has been expanding in health care and may be of benefit [9]. The importance of the role of technology in health care was highlighted by the COVID-19 pandemic with the shift to delivering services via telehealth [10]. Virtual reality (VR) is a digital technology that has become more affordable and accessible, with several studies investigating its use in various health care disciplines and conditions such as posttraumatic stress disorder [11], health professional training [12], and as a distraction tool for patients undergoing chemotherapy [13]. VR is also being explored as a tool for brain injury rehabilitation [14,15] as it may produce benefits over traditional rehabilitation methods [16], such as the ability to control task difficulty [17] and the chance to practice skills in lifelike environments [18], as well as to provide services to patients who experience barriers to accessing care such as cost and geographical isolation [19].

### Social Media in Health Care

In addition to technology, there has been an increasing presence of social networking sites in the health care industry. Uses include disseminating health information [20], enhancing professional networking [21], and collecting data [22]. Twitter (which is now known as X) is one of these social networking sites shown to be a valuable source of public discourse on health care [22]. Established in 2006, Twitter has an estimated 486 million users [23] who send >500 million tweets (ie, messages of up to 280 characters) each day [24]. People “tweet” for various reasons, such as expressing thoughts and opinions, broadcasting news, sharing information, interacting with brands and celebrities, or raising awareness of specific issues [25].

Twitter has been used in health research to better understand behaviors and attitudes toward public health issues to inform health promotion and product development or supplement survey data [22,26-28]. The platform has been used as a data source to determine opinions on organ donation [29]; analyze intentions behind drinking alcohol [30]; communicate public health information [31]; study the tweet content of health professionals [32]; explore the tweets of people with communication disabilities [33,34]; and, more recently, understand the sentiments and experiences related to the COVID-19 pandemic [35].

There are reported benefits of using social media for health-related data collection. Twitter provides access to a large, publicly available data set with the potential representation of geographical and demographic groups that may not usually participate in research [26,36]. Other social media platforms do not offer this same access to data as reciprocal relationships are often needed to view posts and users do not often use hashtags to direct conversations as they do on Twitter. Tweets can also contain unfiltered feedback and user-centric content (eg, personal interests, attitudes, and beliefs), which may not be collected through traditional research methods such as focus groups or surveys because of researchers being present [26,28,37]. In addition, companies are turning to social media as a communication strategy, with Twitter becoming prominent in several contexts (eg, corporate communications and IT marketing) [38]. Information about VR innovation and research is likely to be discussed and released on Twitter given that the platform is popular with IT companies [38], government organizations [20], and think tanks [39]. For these reasons, using Twitter as a data source may offer insights into the uses of VR in health care and brain injury rehabilitation that have not been explored because of VR’s global popularity. Digital discourse and the use of social media increased during the COVID-19 pandemic, with Twitter becoming a source of discussions and information exchange by consumers and thought leaders as scientific conferences [40] and government announcements became digital [20]. Therefore, seeking information about VR user opinions and experiences on Twitter could inform VR development and implementation for brain injury rehabilitation.

### VR Social Media Research

Research on VR using social media platforms is limited. Twitter posts have been explored with regard to VR development [41] and barriers to VR adoption [42]. These studies did not focus on VR in health care but provided useful information about VR trends and potential barriers to use from the business and marketing fields. Shen et al [41] used a concept decomposition approach to analyze Twitter posts about VR in 2015 to 2016. The concept decomposition involved identifying relevant keywords from VR-related tweets and determining the strength of the connections between related terms [41]. The findings demonstrated that tweet content shifted over time, from a focus on VR development to consumer use and applications. Laurell et al [42] used machine learning methods to develop an algorithm that interpreted tweet content related to VR technology, networks, price, and value. The analysis found that Swedish Twitter users viewed VR as becoming more affordable and accessible in 2016 to 2017 yet perceived that VR offered little value for price. The authors concluded that improvements in VR technology may reduce barriers to uptake [42].

Public perceptions of VR in health care have been investigated via the analysis of Facebook user sentiments toward a video depicting VR use in a hospital setting [37]. A total of 1614 public Facebook comments were analyzed from 1 day in March 2016. Most comments were positive in sentiment, and Facebook users offered suggestions for using VR in health care (eg, distraction from pain and decreasing stress). However, some Facebook users expressed potential concerns such as cost, accessibility, VR sickness, and the need for further research [37]. Although this study offered insights into opinions and potential issues with using VR in health care, it was limited in scope.

### User-Based Opinions on and Experiences of VR

Seeking user opinions is essential for successful VR design [43] and achieving effective implementation in clinical practice [37]. End users of VR for brain injury rehabilitation have reported benefits or positive aspects (eg, it being interesting and simple to use) as well as challenges (eg, hardware issues) [44,45]. However, there is an overall paucity of literature related to user opinions and experiences [15]. To address this gap, an alternative, novel approach to collecting data is to interrogate a publicly available data source such as Twitter. Collecting and analyzing Twitter content presents an opportunity to explore user-based discussions on this topic at a large scale and in real time [26]. Unsolicited and unfiltered Twitter conversations could yield potentially untapped demographics, user-centric content, and in-the-moment user experiences or complaints related to VR. Exploring Twitter user networks may help identify key VR end user groups (eg, @users with brain injuries, health professionals, and VR stakeholders), their connections and interactions, and Twitter community characteristics. Exploration of Twitter conversations and users may further inform opinions and experiences related to VR in brain injury rehabilitation and determine whether user experiences in Twitter conversations confirm or differ from those reported in research-based experiences.

### Objectives

Twitter offers a data source that can supplement traditional forms of academic inquiry, such as systematic reviews, particularly in a field that is in its infancy. A hashtag study of VR-related tweets to explore uses and perceptions, particularly in health care and brain injury rehabilitation, has not yet been conducted. Analysis of VR-related tweets may inform design considerations, identify current or potential future uses of VR in health care and brain injury rehabilitation, and assist those interested in the topic in navigating relevant information and networks on Twitter. Therefore, this study aimed to collect and analyze tweets containing the hashtags #VR and #VirtualReality to (1) explore the VR tweeting community to determine topics of conversation and network connections, (2) understand user opinions on and experiences of VR, and (3) identify tweets related to VR use in health care and brain injury rehabilitation.

## Methods

### Ethical Considerations

Ethics approval for this study was granted by the University of Sydney Human Research Ethics Committee (2020/425). Tweet content was paraphrased during reporting of the results to ensure that the content could not be traced to specific Twitter @users owing to the capacity of Twitter and internet search engines to locate matching text [46,47]***.***

### Data Collection

VR-related tweets were identified using a systematic Twitter search process [33,34,48-53]. Tweets were identified using the Twitter search bar and included those posted by @users who set their accounts to public, meaning that their tweets are available to anyone who accesses Twitter [54]. The Twitter search period and tweet capture ran from July 10, 2020, to August 18, 2020 (before the platform was rebranded as X). New tweets containing the hashtag #VirtualReality were sourced once per week, and tweets containing the hashtag #VR were sourced twice per week during this time frame to capture the most relevant tweets*.* The authors did not post any tweets with the hashtags #VR or #VirtualReality during the data collection period. Including additional hashtags such as #BrainInjury, #TBI, #ABI, or #health would have limited the scope for gathering tweets related to user opinions and experiences that would be valuable for consideration in VR development and implementation. A broad approach was also undertaken so as to understand VR-related activity across the platform and provide context for its use.

There are no current recommendations for minimum data sets for a Twitter content analysis [55], although hashtag studies in health care with <3000 tweets have produced valuable results [48]. The time frame and frequency of data collection for a meaningful sample of tweets (ie, related to user experiences and opinions and VR in health care and brain injury) were set a priori based on exploratory Twitter searches by the authors to ensure a sample of at least 3000 meaningful tweets.

Tweets were captured using the NCapture program (QSR International) [56]. Data were imported to the NVivo software (version 12; QSR International) [57] and then exported to an Excel (Microsoft Corp) spreadsheet [58] for analysis. Tweets in the data set were reviewed and excluded based on the following criteria: (1) outside the specified date range, (2) duplicate tweets, (3) not written in English, (4) not related directly to VR (eg, tweets specifically referring to artificial intelligence, augmented reality, or mixed reality; #VR referring to other acronyms such as *voluntary redundancy* or *vocational rehabilitation*; or not referring to immersive VR [59]), (5) tweets identified as having no content (eg, a series of hashtags without meaning), (6) tweets posted by bot @users (ie, @users identified as bots via their username or biographical details), and (7) spam tweets (eg, the same tweet posted by the same @user multiple times in 1 week or identical tweets posted by different @users).

### Data Analysis

This study used established mixed methods for analyzing tweet and Twitter network data [48-50,60].

#### Quantitative Analysis

Quantitative analysis examined the (1) number of tweets, (2) tweet type (ie, original or retweet), and (3) number of unique individuals tweeting about VR. The results are presented descriptively.

#### Content Analysis

All original tweets were analyzed using inductive content analysis [48,51]. The first author assigned codes to each tweet to broadly describe the content that was shared and discussed among the VR tweeting community. If tweets contained insufficient information, the @user’s biographical information and any annotated media or pass-along content were reviewed to provide context. The first and second authors met several times to reach a consensus on coding categories and definitions to enhance trustworthiness [61]. All the authors were involved in discussions related to the final coding categories. Paraphrased tweets were included in the presentation of the results to provide context and clarity for the identified categories.

#### Dann’s Content Classification

Dann’s content classification [50] is a framework for categorizing tweets in a consistent manner and provides insights into how individuals and groups use Twitter for social media communication according to five broad categories: (1) conversational (ie, tweets directed toward other Twitter @users), (2) news (eg, news events, current affairs, or events in progress), (3) pass-along (ie, sharing content or information via internet links or retweets), (4) social presence (ie, greeting or connecting with other @users), or (5) status broadcast (ie, expression of thoughts, experiences, and feelings) [50]. Owing to the large number of tweets collected in this study, only those coded as “VR in health care” and “talking about VR” were analyzed by the first author using Dann’s content classification [50]. The second author independently coded 19.99% (912/4562) of randomly selected tweets from this sample for coding reliability. The point-by-point reliability was 97.6%, and the discrepancies were resolved through consensus discussion. Tweets coded as “VR in health care” and “talking about VR” were also reviewed by the first author for additional content (eg, images, videos, and external URLs) to further describe tweet content and the sharing of information about VR.

#### @User Analysis

The biographical statements of @users tweeting about “VR in health care” and “talking about VR” were examined to characterize @users and provide information about VR Twitter networks. The results are presented descriptively.

#### Computational Analysis

Computational analysis was used to supplement @user and network data. The Gephi software [62] provided a visual representation of the Twitter network data to demonstrate network dimensions and connections between @users who tweeted about VR [51]. Through the use of data visualization, Gephi analysis makes the relationships and communication between Twitter @users visible. Tweet data can be used to generate a graphic representing the network, in which the Twitter @user is at the center (referred to as a “node”) and the communication paths of tweets to and from that @user are represented by the curved lines between 2 nodes (referred to as an “edge”) [49]. Therefore, Gephi visualizations show communication paths between @users (nodes), and they also display tweet communication paths that travel to “the world” (ie, tweets undirected to another @user). A thin line indicates limited interaction between @users, whereas a thicker line represents more frequent communication. A Fruchterman-Reingold layout from Gephi was used given the large network data sets involved [63], with results presented visually and descriptively.

### Methodological Rigor

#### Reporting Framework

This study was guided by the Good Reporting of a Mixed Methods Study [64] (Multimedia Appendix 1 [64]) guidelines.

#### Positionality Statement

At the time of this study, the first author was a Doctor of Philosophy candidate and qualified speech-language pathologist with clinical and research experience in brain injury rehabilitation. The remaining authors were academic researchers with a background in speech-language pathology or psychology. All authors formed part of a research team with expertise in communication disorders following brain injury and applying VR and other technologies to support rehabilitation. The authors MB, EP, AC, and LT also had previous experience in social media research.

## Results

### Overview

**Figure 1:**
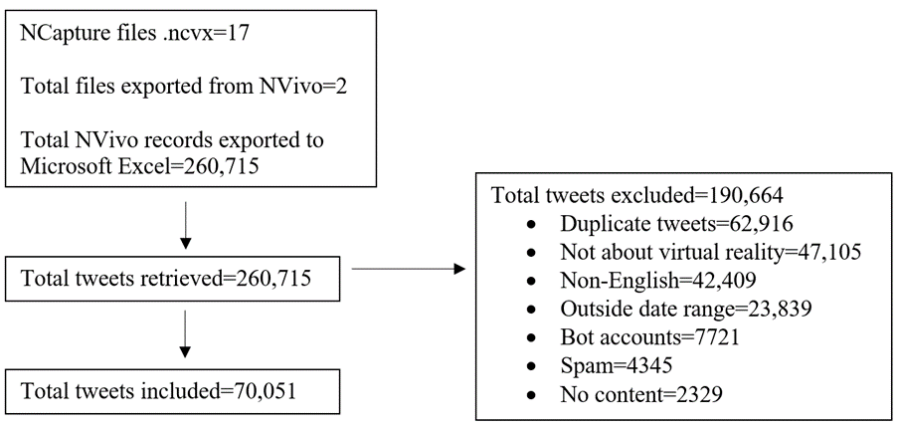
Flowchart of the Twitter search process.

The Twitter search process captured 260,715 tweets containing the hashtags #VR (n=66,974, 25.69%) or #VirtualReality (n=193,741, 74.31%) that were posted between July 10, 2020, and August 18, 2020. Following data collection, all tweets were reviewed for inclusion (shown in Figure 1). A total of 70,051 tweets met the inclusion criteria and were analyzed, which comprised 23,596 (33.68%) original tweets (including quote tweets, in which @users add to another @user’s original tweet) and 46,455 (66.32%) retweets. There were 26,001 unique Twitter @users who posted the 70,051 included tweets.

### Content Analysis

#### Overview

Content analysis of the original tweets (23,596/70,051, 33.68%) revealed that @users tweeted about a broad range of uses, content, news, ideas, and opinions related to VR (Table 1). Most of the included tweets advertised VR products or companies (4176/23,596, 17.7%), followed by tweets with VR content such as screen captures or video playthroughs (3571/23,596, 15.13%). Other tweets were coded as talking about VR (3506/23,596, 14.86%), VR news (3254/23,596, 13.79%), general technology (2628/23,596, 11.14%), VR in industry (2569/23,596, 10.89%), VR live streams (1849/23,596, 7.84%), VR in health care (1056/23,596, 4.48%), VR events (700/23,596, 2.97%), and VR community (287/23,596, 1.22%).

**Table 1:** Content categories of original tweets (N=23,596).

Category	Tweets, n (%)
Advertising and promotion	4176 (17.7)
VR^a^ content	3571 (15.13)
Talking about VR	3506 (14.86)
VR news	3254 (13.79)
General technology	2628 (11.14)
VR in industry	2569 (10.89)
VR live streams	1849 (7.84)
VR in health care	1056 (4.48)
VR events	700 (2.97)
VR community	287 (1.22)

^a^VR: virtual reality.

Tweets coded as “VR in health care” and “talking about VR” were examined in further detail to provide insights into VR that may be useful for VR development and the context in which VR was being applied in health care and brain injury rehabilitation. Additional details related to the original tweet content categories are presented in Multimedia Appendix 2.

#### Content Analysis of “VR in Health Care” Tweets

##### Overview

Original tweets coded as “VR in health care” (1056/23,596, 4.48%) were further classified using content analysis [48,51] to explore applications of VR in the industry. Most tweets were related to uses or potential uses of VR in various disciplines and for various health conditions.

The use of VR simulations in health education and training was mentioned in 272 tweets, with the most frequent uses or potential applications being in medical and surgical training and staff upskilling (n=219, 80.5%), followed by nursing (n=40, 14.7%), dentistry (n=11, 4%), and allied health (n=2, 0.7%). A total of 23.48% (248/1056) of tweets included information on VR use in the areas of psychology, mental health, and cognition. Many of these tweets were related to the use or potential use of VR to treat conditions such as anxiety, posttraumatic stress disorder, and phobias. There were 17.52% (185/1056) of “VR in health care” tweets related to the general use of VR in the health care industry. Some tweets provided information on VR for pain management, distraction from medical procedures, and end-of-life care (108/1056, 10.2%). Other tweets were coded as brain injury rehabilitation (45/1056, 4.3%), neurodegenerative conditions (30/1056, 2.8%), general rehabilitation (29/1056, 2.7%), 3D modeling and medical image viewing (26/1056, 2.5%), visual impairments (24/1056, 2.3%), senior care (23/1056, 2.2%), and other (66/1056, 6.3%). Further information about tweets related to VR in health care is presented in Multimedia Appendix 3.

##### @User Experiences of VR in Health Care

A total of 5.3% (56/1056) of “VR in health care” tweets provided details about @user experiences, commentary, and opinions related to VR in health care. Of these 56 tweets, there were 12 (21%) related to direct @user experiences, including educating medical, nursing, or allied health students (n=7, 58%); distraction from medical procedures (n=2, 17%); pain management (n=2, 17%); and learning about infection control (n=1, 8%). Some @users tweeted to share positive opinions on VR use in health care (19/56, 34%), discuss the use or potential uses of VR in health care (12/56, 21%), network with others using VR in related fields (8/56, 14%), share plans to use VR in health research (1/56, 2%), and express that VR design in health needs user engagement (1/56, 2%). In total, 3 @users expressed challenges with VR: programming difficulties with developing a VR nursing platform, nausea when using a VR exposure therapy platform, and hand tremors affecting VR use by causing shaking and reduced accuracy of gameplay. None of these @users identified as having a brain injury.

##### Sharing Information and Resources

Considering the large proportion of conversational tweets, there were not many discussions between @users about VR in health care. Many conversational tweets contained references to @users or referred content to other @users by tagging them in a tweet. The 1056 original tweets about “VR in health care” were further examined to determine what content was being shared. Most tweets (450/1056, 42.61%) provided a link to a web article or blog that provided information about the use or potential use of VR in health care. Others shared links related to web-based events such as webinars or conferences (117/1056, 11.08%), research articles (111/1056, 10.51%), images or photos (93/1056, 8.81%), videos that demonstrated the use of VR or VR screen recordings (89/1056, 8.43%), websites (36/1056, 3.41%), podcasts (11/1056, 1.04%), research participation links (10/1056, 0.95%), VR app downloads (7/1056, 0.66%), web-based reports (3/1056, 0.28%), a product brochure (1/1056, 0.09%), and a web-based message board (1/1056, 0.09%). There were 6.34% (67/1056) of quote tweets and 5.68% (60/1056) of tweets that contained a broken URL or no additional content.

##### VR in Brain Injury Rehabilitation

A total of 4.26% (45/1056) of “VR in health care” tweets provided insights into VR applications for brain injury rehabilitation. Of these 45 tweets, there were 17 (38%) containing information about VR use specific to stroke rehabilitation (eg, gait, vision, and upper limb retraining). Some tweets (11/45, 24%) provided news updates about companies that make VR products for neurorehabilitation. A VR educational platform for concussion was released during the tweet capture period and was the topic of 20% (9/45) of the tweets. In total, 4% (2/45) of the tweets referenced VR rehabilitation programs for brain injuries caused by various etiologies, including for TBIs. Other tweets included details about VR research for acquired communication disorders (2/45, 4%), web-based events (2/45, 4%), testing a VR neurorehabilitation platform (1/45, 2%), and a VR platform for eye tracking in athletes with brain injuries (1/45, 2%). Most tweets in this subcategory (42/45, 93%) shared links to articles or blog posts (27/42, 64%), journal articles (5/42, 12%), videos (4/42, 10%), quote tweets (4/42, 10%), web-based event registrations (1/42, 2%), and research participation information (1/42, 2%).

#### Content Analysis of “Talking About VR” Tweets

##### Overview

Original tweets coded as “talking about VR” (3506/23,596, 14.86%) included commentary, discussions, opinions, and questions related to @users’ experiences with VR. These tweets were further coded into three subcategories: (1) general conversations about VR (2904/3506, 82.83%), (2) seeking advice or opinions (439/3506, 12.52%), and (3) @user preferences and feedback about VR experiences (163/3506, 4.65%). Additional information about these subcategories is provided in Multimedia Appendix 4.

Almost half of these tweets (1704/3506, 48.6%) shared content related to VR: images or photos (930/1704, 54.58%), videos (427/1704, 25.06%), web-based articles or blogs (177/1704, 10.39%), web-based message boards (118/1704, 6.92%), websites (21/1704, 1.23%), VR live streams (12/1704, 0.7%), VR app store links (10/1704, 0.59%), research articles (4/1704, 0.23%), web-based events (3/1704, 0.18%), and podcasts (2/1704, 0.12%). Content included links to other social media platforms such as YouTube, Reddit, and Instagram. There were 38.36% (1345/3506) of these tweets with no links or media, 11.32% (397/3506) of quote tweets, and 1.71% (60/3506) of broken URLs that were inaccessible at the time of data analysis.

##### General Conversations About VR

There were 82.83% (2904/3506) of “talking about VR” tweets in this subcategory. Many of these tweets (1229/2904, 42.32%) contained details, commentary, and @user views on VR experiences and purchases. @Users also commented on VR news (eg, game releases or updates and VR equipment) and content (554/2904, 19.08%). Tweets also included general commentary and discussions on VR (520/2904, 17.91%). Some @users discussed ideas for VR games or experiences and the potential of VR and its future (295/2904, 10.16%). These discussions focused on updates to existing VR applications, discussions about cross-platform content such as sports and concert viewing, and VR uptake in the context of the COVID-19 pandemic (eg, applications in industries such as education or remote work). Other @users provided insights into VR development processes and shared related images and videos (220/2904, 7.58%). There were also conversations about VR industry challenges (86/2904, 2.96%), such as obstacles to realizing the potential of VR and barriers to entering the industry (eg, cost, usefulness, and access).

##### Seeking Advice and Opinions on VR

These tweets (439/3506, 12.52%) contained questions through which @users sought advice or opinions related to aspects of VR, including suggestions or opinions on VR games and head-mounted displays (HMDs; 147/439, 33.5%). Some @users asked about VR features and development, such as hand tracking, recording, haptics, multiplayer experiences, graphics cards, VR setup, and VR design software (103/439, 23.5%). @Users also requested advice to address VR issues (63/439, 14.4%). Others sought opinions or suggestions related to the VR industry (50/439, 11.4%) and potential uses of VR (24/439, 5.5%).

##### @User Preferences and Feedback on VR Experiences

These tweets (163/3506, 4.65%) provided insights into @users’ VR experiences and preferences that could be useful for informing VR development and associated considerations (eg, design aspects, hardware and software issues, adverse effects, and accessibility). Of these 163 tweets, 55 (33.7%) were related to the potential adverse effects of VR use, such as VR sickness and discomfort when wearing HMDs (eg, nausea, headache, dizziness, and sore eyes). Some @users provided information about ways to minimize adverse effects (eg, starting with seated VR experiences or using HMD comfort modifications), whereas others described a lack of adverse effects. Other tweets (50/163, 30.7%) contained information or solutions related to issues that @users experienced with VR (eg, hardware issues, audio or graphic glitches, connectivity issues, and gameplay error messages). The tweets also contained @user preferences and opinions regarding aspects of VR design (32/163, 19.6%), including discussions about hardware, field of view, latency, scaling, and wireless capabilities. VR accessibility was discussed in 16% (26/163) of tweets, in which @users tweeted about the need to make VR accessible to people who have visual impairments or physical limitations or who are deaf or hard of hearing. Some @users described VR accessibility options such as closed captions, single controller use, and prescription lenses.

### Dann’s Content Classification

#### Overview

Tweets within the categories “VR in health care” and “talking about VR” were coded based on Dann’s content classification [50] (Table 2).

**Table 2:** Dann’s content classification [50] of original tweets within the “VR^a^ in health care” and “talking about VR” categories (N=4562).

	“VR in health care” tweets (n=1056), n (%)	“Talking about VR” tweets (n=3506), n (%)
Conversational	434 (41.1)	1361 (38.82)
Pass-along	561 (53.13)	805 (22.96)
News	29 (2.75)	9 (0.26)
Status broadcast	25 (2.37)	942 (26.87)
Social presence	7 (0.66)	389 (11.1)

^a^VR: virtual reality.

#### “VR in Health Care” Tweets

Most tweets (561/1056, 53.13%) in this category were coded as pass-along, followed by conversational (434/1056, 41.1%), news (29/1056, 2.75%), status broadcast (25/1056, 2.37%), and social presence (7/1056, 0.66%) tweets. In the subcategory “brain injury rehabilitation” (45/1056, 4.26% of tweets), of the 45 tweets, there were 27 (60%) pass-along tweets and 18 (40%) conversational tweets.

#### “Talking About VR” Tweets

Original tweets in this category were classified as conversational (1361/3506, 38.82%), followed by status broadcast (942/3506, 26.87%), pass-along (805/3506, 22.96%), social presence (389/3506, 11.1%), and news (9/3506, 0.26%).

### Twitter @User Analysis

#### Overview

The biographical details of @users who tweeted within the categories of “VR in health care” and “talking about VR” were examined to inform @user networks (Table 3).

**Table 3:** @User groups for original tweets within the “VR^a^ in health care” and “talking about VR” categories (N=2679).

User group	@Users posting “VR in health care” original tweets (n=622), n (%)	@Users posting “Talking about VR” original tweets (n=2057), n (%)
VR, technology, or gaming companies	123 (19.8)	191 (9.3)
Health care or health technology professionals and researchers	118 (19)	19 (0.9)
@Users with an interest or expertise in VR, gaming, or technology	105 (16.9)	1195 (58.1)
Health technology companies	73 (11.7)	4 (0.2)
Health organizations	64 (10.3)	0 (0)
Health, technology, or gaming news outlets	33 (5.3)	18 (0.9)
No or insufficient biographical data	32 (5.1)	260 (12.6)
Organizations and companies unrelated to health care or VR	25 (4)	37 (1.8)
General public	21 (3.4)	329 (16)
Twitter accounts for conferences or expositions	12 (1.9)	1 (0)
News outlets unrelated to VR or health	9 (1.4)	3 (0.1)
Academic journals	7 (1.1)	0 (0)

^a^VR: virtual reality.

#### @Users Tweeting About “VR in Health Care”

There were 2124 unique @users who posted an original tweet, retweet, or both about VR in health care: 622 unique @users posted the 1056 original tweets, and 1599 @users posted 2733 retweets. The biographical statements and usernames of the @users who posted original tweets were examined and coded. The largest group of @users who posted original tweets about “VR in health care” were VR, technology, or gaming companies (123/622, 19.8%). The next largest group included health care or health technology professionals and researchers in the fields of medicine, nursing, and allied health (118/622, 19%). The remaining categories of @users included people with an interest or expertise in VR, gaming, or technology (105/622, 16.9%); health technology companies (73/622, 11.7%); health organizations (64/622, 10.3%); health, technology, or gaming news outlets (33/622, 5.3%); no or insufficient biographical data (32/622, 5.1%); organizations and companies unrelated to health care or VR (25/622, 4%); the general public (21/622, 3.4%); Twitter accounts for conferences or expositions (12/622, 1.9%); news outlets unrelated to VR or health (9/622, 1.4%); and academic journals (7/622, 1.1%). Of the 622 @users tweeting about VR in health care, 43 (6.9%) posted original tweets about VR use in brain injury rehabilitation. Health care professionals accounted for 23% (10/43) of these @users.

#### @Users Tweeting About “Talking About VR”

A total of 3752 unique @users posted an original tweet, retweet, or both within the category “talking about VR”: 2057 @users posted the 3506 original tweets, and 1857 @users posted 2782 retweets. Most @users in this category who posted original tweets (1195/2057, 58.09%) identified as having an interest or expertise in VR, gaming, or technology. Other @users were categorized as the general public (329/2057, 15.99%); no or insufficient biographical data (260/2057, 12.64%); VR, technology, or gaming companies (191/2057, 9.29%); organizations and companies unrelated to VR or technology (37/2057, 1.8%); health care or health technology professionals (19/2057, 0.92%); VR or technology news outlets (18/2057, 0.88%); health technology companies (4/2057, 0.19%); news outlets unrelated to VR or technology (3/2057, 0.15%); and a Twitter account for a conference (1/2057, 0.05%).

### Computational Analysis

**Figure 2:**
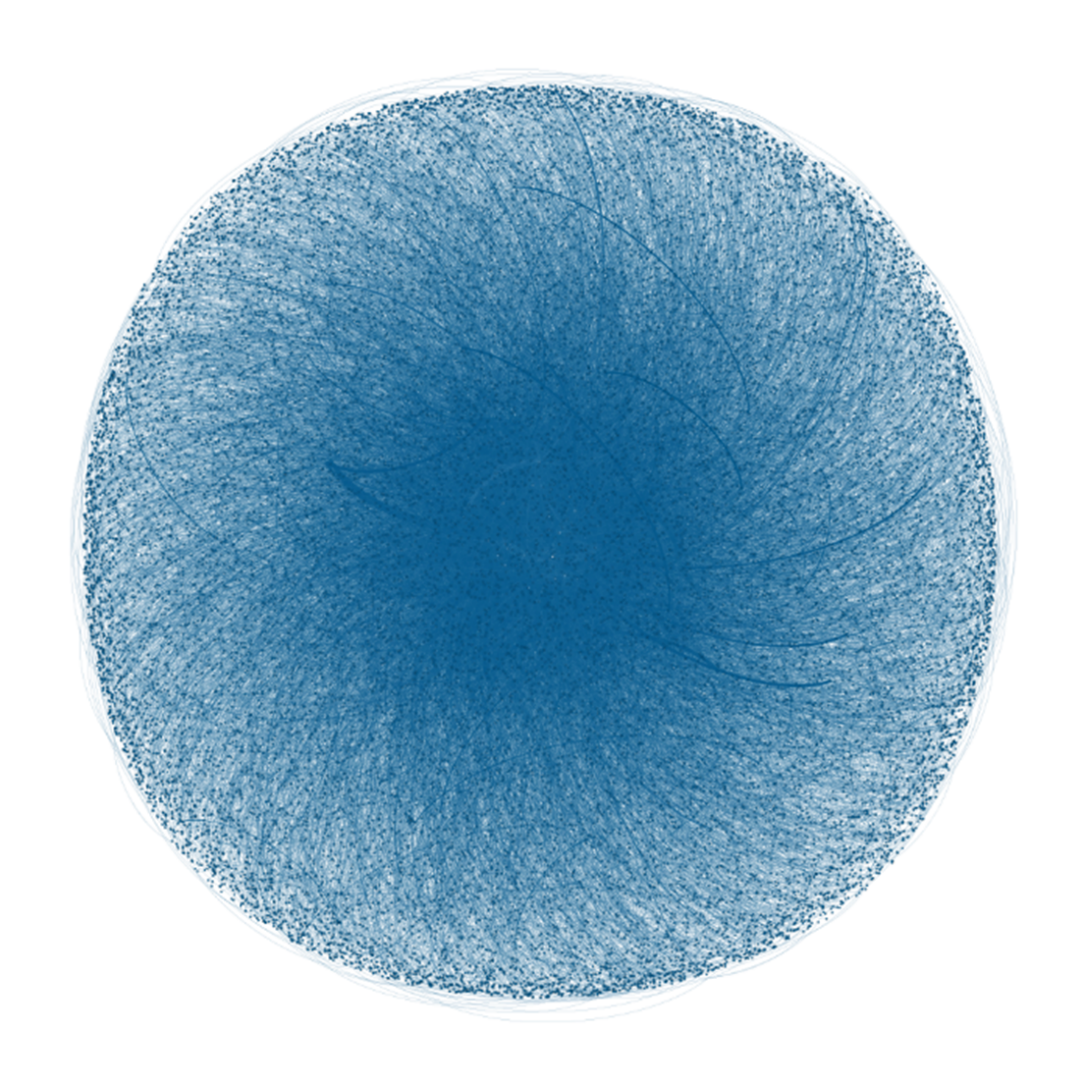
Gephi network visualization of the virtual reality tweeting community.

A Gephi visualization of the 26,001 @users who tweeted or retweeted posts containing the hashtags #VR and #VirtualReality is shown in Figure 2. The visualization shows that (1) a large, dense network of @users discussed VR, with some tweeting more than others (ie, evidenced by the thicker, darker lines between nodes); (2) most of the tweets were undirected (ie, not sent to specific @users but sent out to the Twitter void); and (3) many @users were not connected to one another within a central network.

**Figure 3:**
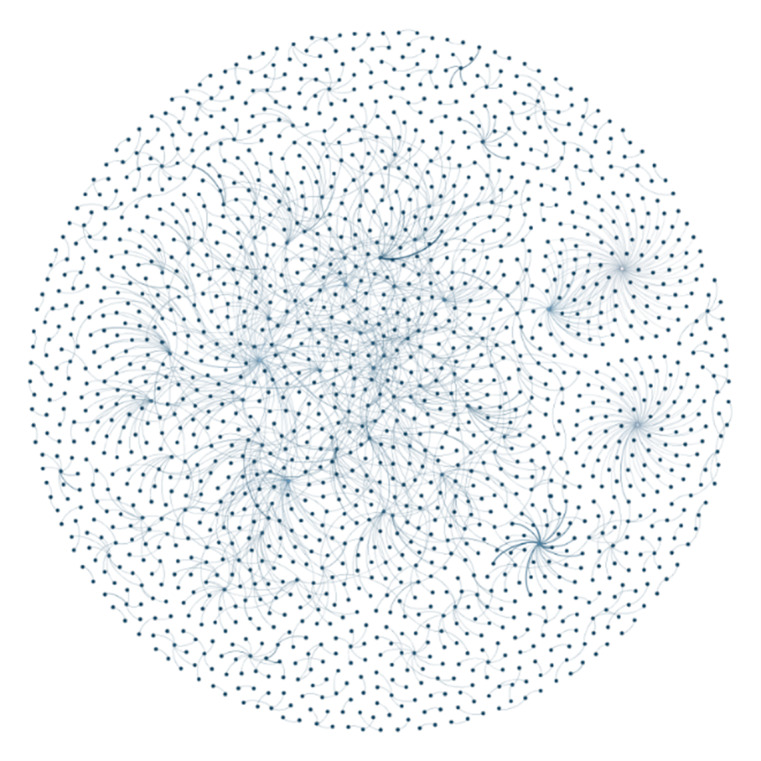
Gephi network visualization of the tweeting community for the “VR in health care” category.

Figure 3 shows the network of 2124 @users who posted tweets coded as “VR in health care.” This visualization demonstrates that (1) overall, there were many individual @users sending tweets who were not necessarily connected with others in the network; (2) some individuals were connected within smaller central networks, but they were not highly interconnected; and (3) a small number of @users were connected to others on the periphery and were separate from the more central @users and small networks.

**Figure 4:**
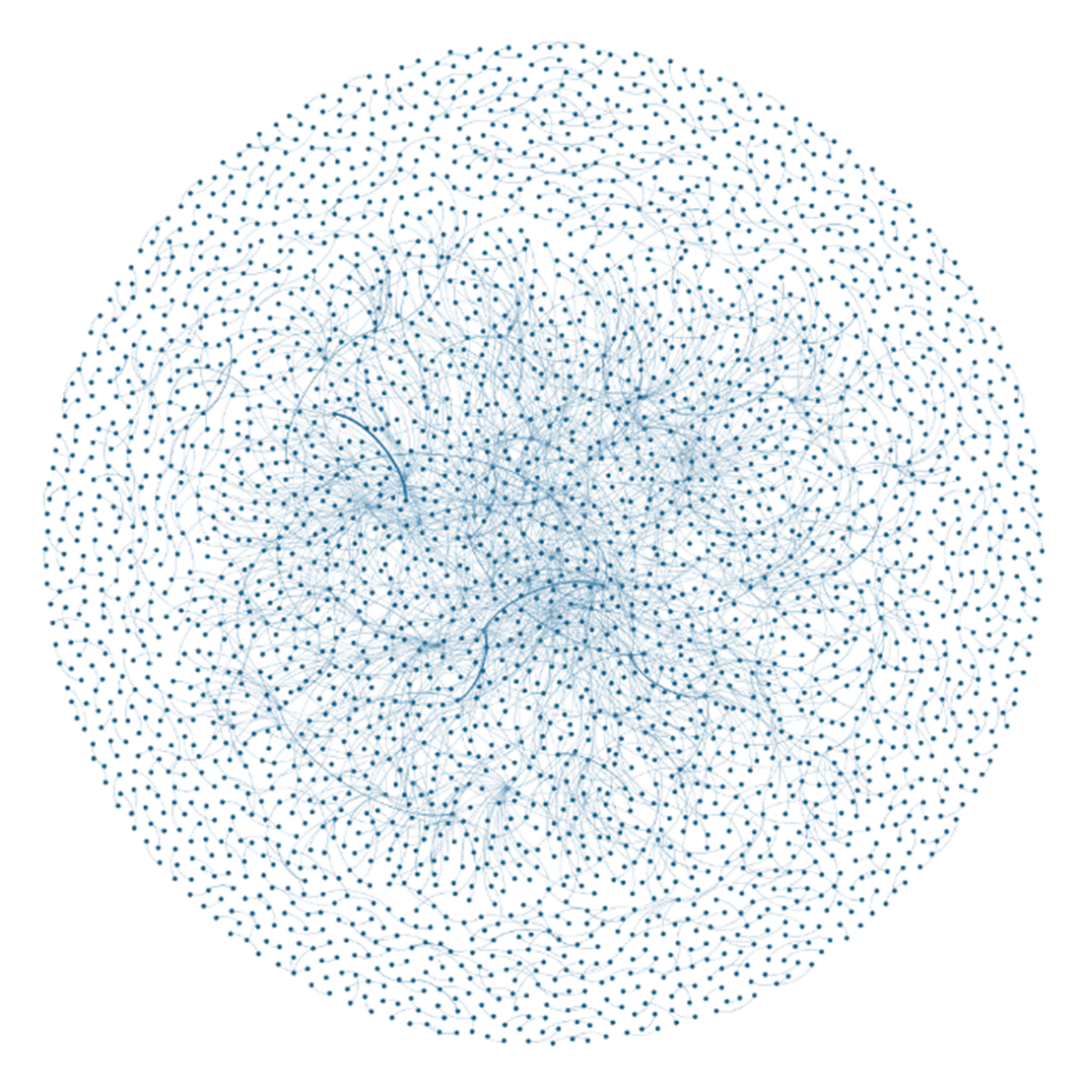
Gephi network visualization of the tweeting community for the “talking about VR” category.

A visualization of the network tweeting within the category “talking about VR” is presented in Figure 4 (n=3752 @users). Within this network, there were (1) many @users connected within a central community, (2) numerous outliers that were not connected to the central community, and (3) no distinct small VR tweeting networks.

## Discussion

### Principal Findings

#### Overview

This study explored tweets about VR that were posted over a 6-week period from July 2020 to August 2020. The analysis of tweets containing the hashtags #VR and #VirtualReality provided an overview of VR-related topics, conversations, and @user networks on Twitter. Applications of VR in health care and brain injury rehabilitation were also explored.

A total of 10 main content themes were identified via a content analysis of 23,596 original tweets. In this tweet sample, @users predominantly tweeted to advertise VR-related content and products such as applications and hardware. This finding was not surprising given the increased use of social media platforms for driving digital engagement with brands [65]. Twitter is also evidently a social networking site that people with an interest in VR use to share VR-related content, experiences, and news. This finding reflects general Twitter use trends as news is frequently distributed via the platform in addition to it being a platform for conversing and sharing ideas [66,67]. Tweet content also reflected VR applications in different industries, including education [68], real estate [69], tourism [70], and architecture [71].

Tweets were typically related to events or news that occurred at the time of data collection. The COVID-19 pandemic may have led to a focus on VR use in industries, discussion of VR ideas and potential owing to stay-at-home directives, and the necessity of technology to keep people entertained and connected. Similar trends have been noted in tweets related to the use of other technologies and telepractices during the pandemic [72,73]. It is important to note that 260,715 tweets containing the hashtags #VR and #VirtualReality were collected, yet only 70,051 (26.87%) were eligible for analysis. Previous research has suggested that using Twitter as a data source may harvest irrelevant data, especially as tweets are typically unprompted and not created to address research purposes [73]. This was confirmed in this data set, in which >150,000 tweets included irrelevant content, were posted by bot accounts, or included spam content. As such, navigating content on Twitter may be challenging for users to easily find relevant information about VR experiences or best practices for rehabilitation.

#### The VR Tweeting Community

The Gephi visualizations (Figures 2-4) provided an understanding of the communities engaging in VR discussions. A large network of @users formed the overall VR tweeting community, although many were not interconnected and there were no clear central networks. The visualization (Figure 2) also showed that, although a proportion of @users tweeted several times, most sent one-off tweets within the collected sample. Within the “VR in health care” subset, a small number of @users were connected (Figure 3). However, these connections were not demonstrated across the network and included smaller groups of @users. In comparison, the “talking about VR” network had a larger and more connected central community. This finding indicates that, although there may be smaller networks of health care professionals with an interest in VR, a larger interconnected Twitter community is yet to be established. An interconnected community could offer a platform to exchange ideas, seek support, and establish networks [74].

Examining the biographical data of @users posting original tweets in the “VR in health care” and “talking about VR” content categories provided insights into the VR tweeting community (Table 3). During the data collection period, Twitter handles of health care professionals; VR companies or products; and those with an interest or expertise in VR, technology, or gaming accounted for a similar number of tweets related to VR use in health care (16%-20% each). However, health professionals made up a small proportion of the overall number of individual Twitter @users identified in this study via user analysis (Table 3).

No @user who tweeted using the hashtags #VirtualReality or #VR during the 6-week data collection period identified as having a brain injury. Previous research suggests that people with TBI use Twitter for various reasons, yet few of their tweets refer to rehabilitation experiences [33,48]. People with brain injuries are underrepresented on Twitter [48], which may be reflected in the findings of this study. If individuals with brain injuries are interested in VR, Twitter could provide a platform for accessing relevant information and examples as well as opportunities to engage with others who are also interested in VR (eg, the potential to connect and establish VR-based social interactions).

@Users with an interest or expertise in VR, gaming, or technology accounted for >50% (1195/2057, 58.09%) of @users within the content category “talking about VR,” and the network analysis (Figure 4) showed a connected group of @users interacting. This finding suggests that Twitter is an important platform for the VR community to share experiences and opinions, comment on news, disseminate events, and seek advice within a supportive community. This could also explain the fact that many tweets had content or additional hashtags referring to technology or gaming terminology that may not be immediately familiar to the broader public who use Twitter (eg, #IoT, #metaverse, #blockchain, #crypto, and #GameDev; Multimedia Appendices 2 and 4). This content might not be accessible to people with brain injuries and associated communication disabilities as they may have difficulty comprehending these terms.

#### VR in Health Care and Brain Injury

A small community tweeted about VR use in health care, with tweets related to this topic accounting for 4.48% (1056/23,596) of the analyzed tweets. Over half (561/1056, 53.13%) of the tweets within this subcategory comprised pass-along tweets, in which @users shared information through web-based news articles, research publications, and web-based events. The analyzed tweets demonstrated that VR is being used in various health care disciplines. Education for medical professionals and psychology were the most represented fields, accounting for almost half (520/1056, 49.24%) of the tweets in the “VR in health care” category. This finding reflects major VR research themes in the literature [75]. The use of VR could be lagging in other fields given these findings, or those using or researching VR in these areas may not be using Twitter to promote their work. Alternatively, the data were a snapshot of tweets collected over a 6-week period, so the included tweets only accounted for news, events, experiences, and publications within or close to this time frame that were shared on Twitter. Expanding the time frame of data collection may have provided further insights into tweets related to VR in health care.

Although conversational tweets accounted for 41.1% (434/1056) of tweets in the “VR in health care” category, @users did not have robust conversations about using VR in the field. For example, @users in this study were tagged in tweets to promote VR applications or share related articles or events, with only 5.3% (56/1056) of tweets referencing a direct experience of or opinion on VR related to health care. Similar studies exploring health-related hashtags on Twitter also found minimal discussion about topics among Twitter networks [76]. This differs from non–health care @users in this study, who shared how they used VR and showcased different VR examples and experiences. In addition, only 0.28% (3/1056) of tweets in this category provided insights into the challenges that people using VR in health care may face, and none of these tweets were posted by @users with a brain injury.

The use of VR in brain injury rehabilitation was identified in only 0.19% (45/23,596) of original tweets. Many of these tweets discussed the use of VR for physical or cognitive impairments rather than for acquired communication disorders and for stroke rather than for TBI rehabilitation. This finding is consistent with the published literature [15] in that the use of VR in these fields is yet to be established and evaluated, in addition to there being limited guidance for its use in clinical practice. There was little research dissemination, reflecting hashtag studies for brain injury that have found a lack of research engagement via Twitter [48]. To date, Twitter research related to brain injury appears to be limited to TBI-related hashtags [48], the use of Twitter by people with TBI [33], a network and content analysis of aphasia-related tweets [76], and tweets about concussion [77-79].

#### @User Opinions and Experiences Related to VR

This study demonstrated that Twitter is an important platform for people and companies with an interest in VR to share experiences, opinions, and content related to VR. Most tweets coded as “talking about VR” were conversational, with the purpose of engaging in discussions or drawing the attention of other @users. The platform was also used to seek advice from other @users with an interest in VR or share opinions and experiences related to VR that were almost always positive. However, these opinions and experiences should be considered in the context of those who use Twitter as many @users had a specific interest or expertise in VR and the platform does not typically represent the views of the general population [36]. Many of these tweets contained links to content hosted on other social media platforms (eg, YouTube, Twitch, Instagram, or Facebook) or web-based articles and blogs. This content could be used by clinicians and those interested in VR to learn about how it works or view examples of VR applications (eg, viewing VR content on YouTube).

It is important to note that some @users highlighted concerns with VR, either commenting directly on issues affecting their VR experience (eg, VR sickness, hardware and software issues, and accessibility) or the VR industry (eg, cost and access). Previous social media [37,42] and end user research [80-83] related to VR barriers has also found that VR sickness, cost, and accessibility were featured in discussions. However, these tweets formed a small proportion of the overall tweet sample in this study (55/23,596, 0.23% of analyzed tweets). Although these issues were not described by people identifying as having a brain injury, the experiences of >2600 Twitter @users provided valuable insights into potential challenges to be aware of and consider when using VR in health care settings. Considerations not previously described in recommendations for VR development for brain injury rehabilitation [15] were identified in this study, which were mainly related to technical aspects of VR (eg, interaction mechanisms, game engines, latency, and image resolution). These design considerations are similar to those for developing VR-based exposure therapy [84].

### Limitations

Although this study analyzed a meaningful sample of tweets, not all tweets containing #VR or #VirtualReality posted during the 6-week sample period were captured and analyzed. This limitation is due to the inherent constraints of the Twitter application programming interface search algorithm, which limits the number of tweets collected at one time. In addition, not all tweets related to brain injury or health and the use of VR were captured and analyzed within this time frame. Tweets sent from private Twitter accounts and those written in languages other than English were also not analyzed. Furthermore, Dann’s [50] content coding and @user analysis were limited to tweets relevant to the aims of this study (ie, coded as “VR in health care” and “talking about VR”) and content coding focused on original tweets (ie, excluded retweets or content in quote tweets). The analysis of more tweets from the data set using these methods may have provided additional information about the VR tweeting community and how the platform is used to disseminate related information and content.

### Comparison With Prior Work

The findings of this study support evidence from the literature that the use of VR in brain injury rehabilitation, particularly for TBI [15], is limited. Important user considerations for VR implementation in health care and brain injury rehabilitation were identified in Twitter @users’ experiences (eg, usability, accessibility, and VR sickness) [15,43], highlighting the need to engage end users in VR design and feasibility testing [43,85] as well as take technical aspects into account. This study showed that valuable insights can be drawn from Twitter, which constituted a novel data source of >2600 unique “voices” that discussed their own experiences of and opinions on VR, demonstrated an interest in VR health applications, and reflected related social media research on VR in health care [37].

### Clinical Implications and Future Directions

Twitter has been identified as a platform that can be used in health care to determine public perceptions, recruit research participants, and share and advance research [20,22,86,87]. There is potential to disseminate VR news, research, and events in this field via social media platforms. However, given the large number of tweets containing the hashtags #VR and #VirtualReality, it may be challenging to navigate and find relevant information on VR use in health care. Including targeted hashtags (Multimedia Appendices 2-4) in Twitter searches may identify more relevant content if clinicians are interested in obtaining information about VR. Exploring VR-related hashtags on other social networking sites could further inform how social media is used to disseminate information about VR in health care and whether different platforms yield different findings.

Researchers and clinicians who use VR could promote their work via Twitter [48,51] or other social networking sites, which may increase interest in and awareness of VR, promote networking opportunities, and showcase potential uses to patients and clinicians. Sharing examples and engaging in discussions could demonstrate how VR could be implemented in clinical practice or ease uncertainties related to its use (eg, VR sickness or suitability for specific patient groups). @Users with a brain injury could also find it challenging to navigate VR-related topics on Twitter as many can experience cognitive and communication difficulties. For example, people with TBI have reported that they tweet for different purposes, including to share their experiences of living with a TBI, support others, and seek information [33]. Therefore, clinicians could support people with brain injuries and associated communication disorders to navigate topics on Twitter [48,76], particularly given the potential concerns related to the use of social media by people with brain injuries (eg, internet safety and confidentiality) [88].

Some tweets provided insights into challenges that VR users may experience, which could apply to using VR in health care settings. Health professionals may anticipate these potential issues and identify ways to overcome them. However, it is important to consider that the reported issues were mostly from @users who did not identify as having a health condition or disability, meaning that their experiences cannot be relied on alone to inform VR design in health care. Exploring the functionality of VR with different patient groups is necessary to determine VR usability [83], including any barriers to using VR in clinical settings or for leisure. This is particularly important for people with a brain injury as they can experience physical, cognitive, and communication impairments that may affect their use of VR [15]. It will be necessary to explore the perspectives of people with brain injuries and their clinicians in relation to VR use as it is recommended that end users be involved in developing VR for health care [43] and brain injury rehabilitation [15]. Interviews or focus groups could be conducted with these key end users to establish their views on using VR in rehabilitation, including perceived barriers and facilitators. Once these barriers and facilitators are established, user-based testing of VR with people with brain injuries and their clinicians should be implemented to ensure that VR applications are targeted to their needs.

### Conclusions

This study explored VR-related tweets and networks with a focus on user experience and applications in health care and brain injury rehabilitation. Content analysis revealed that the VR tweeting community used Twitter for various purposes, including promoting VR-related products, sharing content, disseminating news and events, and talking about experiences and opinions. The platform provided insights into the use of VR in health care, with @users predominantly disseminating research and information on clinical VR applications rather than experiences of or opinions on VR use in this field. Tweets referring to VR in brain injury rehabilitation comprised a small proportion and did not provide an in-depth insight into the use of VR by this population.

Twitter has the potential to showcase VR use in health care and brain injury rehabilitation to promote uptake or disseminate research findings. However, the vast amount of information and potential for unrelated content suggest that related hashtags should be considered when searching for VR content on Twitter. Those involved in developing or using VR in brain injury rehabilitation should also consider the reported issues and challenges identified in the tweet sample, including cost, accessibility, VR sickness, and technical design aspects. Few tweets provided insights into user experiences of and opinions on VR in health settings, with none provided by people with brain injuries. Further research is needed to determine the VR needs and experiences of people with brain injuries and their clinicians to guide the design of VR applications for effective rehabilitation.

## Appendix

Multimedia Appendix 1 GRAMMS (Good Reporting of a Mixed Methods Study) checklist.

Multimedia Appendix 2 Details on original tweet content categories.

Multimedia Appendix 3 Details on original tweets related to “VR in health care.”.

Multimedia Appendix 4 Details on original tweets related to “talking about VR.”.

## Abbreviations

HMD: head-mounted display
TBI: traumatic brain injury
VR: virtual reality

## Data Availability

The data sets generated and analyzed during this study are available from the corresponding author upon reasonable request.
