# Supplementary material for: Exploring Discussions About Virtual Reality on Twitter to Inform Brain Injury Rehabilitation: Content and Network Analysis
Source: J Med Internet Res. 2024 Jan 19;26:e45168. doi: 10.2196/45168 (PMC10837760; doi:10.2196/45168)
Supplement: Multimedia Appendix 1 [file jmir_v26i1e45168_app1.pdf]

Good Reporting of a Mixed Methods Study (GRAMMS) Checklist

Item	Guide questions/description	Location
1	Describe the justification for using a mixed methods approach to the research question	Pages 3-4
2	Describe the design in terms of the purpose, priority and sequence of methods	Pages 3-4
3	Describe each method in terms of sampling, data collection and analysis	Pages 3-4
4	Describe where integration has occurred, how it has occurred and who has participated in it	Pages 3-4
5	Describe any limitation of one method associated with the presence of the other method	Pages 11-14
6	Describe any insights gained from mixing or integrating methods	Pages 11-14

The GRAMMS Checklist is from O'Cathain A, Murphy E, Nicholl J. The quality of mixed methods studies in health services research. Journal of Health Services Research & Policy. 2008;13(2):92-8. doi: 10.1258/jhsrp.2007.007074.

This is a Multimedia Appendix to a full manuscript. For full copyright and citation information see doi: 10.2196/45168.
