# Supplementary material for: Exploring Discussions About Virtual Reality on Twitter to Inform Brain Injury Rehabilitation: Content and Network Analysis
Source: J Med Internet Res. 2024 Jan 19;26:e45168. doi: 10.2196/45168 (PMC10837760; doi:10.2196/45168)
Supplement: Multimedia Appendix 2 [file jmir_v26i1e45168_app2.pdf]

**Details on Original Tweet Content Categories (N=23,596)**

| Coding Category (Number of Original Tweets, %) | Description | Examples of Related Hashtags | Example Tweets (Paraphrased) |
|---|---|---|---|
| Advertising and promotion (n=4176, 17.7%) | Tweets that promoted VR products or companies (eg, headsets, accessories, merchandise, gaming avatars, VR assets, VR product or company websites, VR apps and software downloads, game trailers, books, VR arcades). Also included tweets that included self-promotion or promotion of others in the VR industry, describing VR services and products, and promotion of VR competitions, fundraisers, and giveaways. | #GameAssets<br>#promotions<br>#DigitalMarketing<br>#3Dmodels<br>#SketchFabStore<br>#EarlyAccess<br>#IndieDev<br>#giveaway | Try the new [VR app] released today! #VR<br><br>Check out this deal for 10% off [VR game]! |
| VR content (n=3571, 15.13%) | Tweets that contained VR content without significant commentary (eg, screenshots of a VR scene or character, videos of playthroughs of VR games or VR content, screenshots or videos of VR content in development, videos or internet content that could be viewed in 360 degrees using a VR headset). Videos were often hosted on YouTube. | #animation<br>#3Dmodeling<br>#MadeWithUnity<br>#ScreenshotSaturday<br>#GameDev<br>#GamePlay<br>#VTuber<br>#CharacterDev | See my VR playthrough via #YouTube #VirtualReality #gamers |
| Talking about VR (n=3506, 14.86%) | Tweets where @users discussed VR, provided or sought advice/opinions, general commentary and conversations about VR use and potential, descriptions of using VR, plans to use VR, issues associated with VR design or use. Further details are presented in Multimedia Appendix 3. | #tech<br>#gamer<br>#GameDev<br>#unity3d<br>#IndieDev<br>#VRgaming | Any suggestions for audio glitch with VR HMD? #Oculus #VirtualReality<br><br>So cool!! Cannot wait to play this new #VR game |

| Coding Category (Number of Original Tweets, %) | Description | Examples of Related Hashtags | Example Tweets (Paraphrased) |
|---|---|---|---|
| VR news (n=3254, 13.79%) | Tweets about news related to VR (eg, news about VR games and applications, app reviews, equipment, companies, industry awards, interviews and podcasts with people in the VR industry, general VR information and updates). Many of these tweets included a weblink to a news article or a blog post. | #htcvive #oculus #OculusQuest #gaming #games #technology #news #ARVRnews #VRnews | [VR company] to launch new #VR app [URL]<br><br>Check out my review of this #VR game to be released next week [URL] #VirtualReality #news #gamers #review |
| General technology (n=2628, 11.14%) | These tweets typically contained information about technology-related news, events (eg, webinars, conferences), VR/technology development courses or tutorials, information about uptake of technology and future applications in everyday life and industry. Tweets included in this category also mentioned VR in the context of other technologies and news about technology trends (eg, artificial intelligence, augmented reality, the internet of things, mixed reality, 5G networks, blockchain, cryptocurrency, haptics), VR job advertisements, and VR funding opportunities. | #TechStuff #innovation #IoT #BigData #technology #XR #metaverse #AugmentedReality #ArtificialIntelligence #5G #enterprise #blockchain #crypto #MixedReality #3Dmodel #GameDev | See this list of 10 #tech trends [URL] #AR #AI #VirtualReality #5G #TechForGood<br><br>Check out this webinar on #unity development #VR #3D #gamedev [URL] |

| Coding Category (Number of Original Tweets, %) | Description | Examples of Related Hashtags | Example Tweets (Paraphrased) |
|---|---|---|---|
| VR in industry (n=2569, 10.89%) | Tweets about use or potential use of VR in industries other than health care for training, collaboration or work purposes including in education, real estate, construction, marketing, workplaces, skills training, tourism, automotive industry, emergency services (eg, firefighting, law enforcement), entertainment (eg, theatre, movie viewing, museum viewing), sports viewing, fitness. These tweets typically included a link to a news article or a blog post that provided information about uses or potential uses of VR in industries; some with a focus on the role of VR in workplaces as a result of the COVID-19 pandemic. Research about VR in industries was also included. | #education<br>#school<br>#future<br>#DigitalMarkting<br>#RemoteWork<br>#collaboration<br>#business<br>#innovation<br>#training<br>#VRsolutions | See how #VR could change the #future of work [URL] #VirtualReality #RemoteWork<br><br>VR is being used to train workers [URL] #work #VR #training<br><br>Is VR the next big thing in real estate? [URL] #RealEatste #VirtualReality #sales |
| VR live streams (n=1849, 7.84%) | Tweets that contained a link to a VR live stream (eg, Twitch account). | #twitch<br>#LiveStream<br>#TwitchStreamers<br>#VRgaming<br>#VRgamers<br>#streamer | Ready to stream tonight!!! [Twitch URL] #streamer #twitch #VR #LiveStream |
| VR in health care (n=1056, 4.48%) | Any tweet related to the use of VR in health care settings (eg, online events, articles, videos, research papers). Further details are presented in Multimedia Appendix 3. | #MedTech<br>#Healthcare<br>#rehabilitation<br>#DigitalHealth<br>#simulation<br>#MedSim<br>#HealthTech | #VirtualReality is being used in the treatment of #PTSD. Check out this new research article #HealthTech #VR #Psychology |

| Coding Category (Number of Original Tweets, %) | Description | Examples of Related Hashtags | Example Tweets (Paraphrased) |
| --- | --- | --- | --- |
| VR events (n=700, 2.97%) | Tweets that promoted or provided information about events held in VR or events about VR (eg, online conferences or webinars about VR only). Tweets often included links to event registration, articles/blogs promoting VR-related events, or videos of VR events. Events included webinars on VR design, conferences about VR in use in industries, VR exhibitions, VR music concerts and meet-ups in VR. | #OnlineEvent<br>#Event<br>#SocialVR<br>#AltSpace<br>#VirtualEvents | Sign up for next week's online event about #VR [URL]<br><br>Meet up in #VR! Rave at 8pm ET TONIGHT! See schedule here [URL] #live #meetup #event #fun |
| VR community (n=287, 1.22%) | Tweets where VR users used Twitter to connect with other VR users (eg, thanking for attending a VR livestream, thanking subscribers, recommending other @users to follow or subscribe to, invitations to Discord groups or events, congratulating on VR-related achievements). These tweets did not contain discussions about specific VR experiences or issues, but rather fostered a sense of connection within the VR tweeting community. | #VRgamers<br>#VRcommunity<br>#VRchat<br>#GamingCommunity<br>#VRpeeps<br>#GameDev<br>#GameDeveloper | Thanks for attending my #VR stream last night! Hope u have a fantastic day! #stream #VRpeeps<br><br>Huge congrats [@user] on your game release! #GameDev #VR |

This is a Multimedia Appendix to a full manuscript. For full copyright and citation information see doi: 10.2196/45168
