# Supplementary material for: Exploring Discussions About Virtual Reality on Twitter to Inform Brain Injury Rehabilitation: Content and Network Analysis
Source: J Med Internet Res. 2024 Jan 19;26:e45168. doi: 10.2196/45168 (PMC10837760; doi:10.2196/45168)
Supplement: Multimedia Appendix 3 [file jmir_v26i1e45168_app3.pdf]

Details on Original Tweets Related to 'VR in Health Care' (N=1056)

Coding Category (Number of Original Tweets, %)	Number of @Users	Category Description	Example Tweet (Paraphrased)
Education and training for health care professionals (n=272, 25.76%)	181	Tweets related to the use of VR for training or education of medical students and doctors (n=219) (including development of empathy, diabetes education, advance life support training, COVID-19 related training, infection control practices, surgical training/preparation, radiology, ophthalmology, first responders, intubation, de-escalation of aggression). Many of these tweets were showcasing or advertising VR platforms for medical educations. This category also included tweets about VR education and training for nursing students and clinicians (n=40), dentistry (n=11), physical therapy (n=1) and speech-language pathology (n=1). Some tweets also promoted VR medical/surgical simulation platforms.	Read this article to learn how #VR can help #learning in #MedicalSchool<br><br>The future of #surgery is in #VirtualReality #tech #healthcare<br><br>Our #VR sim platform can be used to prepare #doctors in emergencies. Check it out here [URL] #tech #MedSim #HealthTech #training
Mental health, psychology and cognition (n=248, 23.48%)	173	Tweets about the use of VR to manage mental health or psychological conditions (n=210) (eg, anxiety, post-traumatic stress disorder, social anxiety, phobias, addiction recovery, COVID related stress and anxiety, obsessive compulsive disorder), and in cognition (n=38), including for navigational efficiency, memory, attention, influence on memory formation, social cognition, attention (typically in healthy populations rather than treating people who have cognitive impairments).	#VR is being used to treat PTSD. Read more here [URL]<br><br>New article out about use of #VirtualReality to treat #stress and #anxiety [URL]

Coding Category (Number of Original Tweets, %)	Number of @Users	Category Description	Example Tweet (Paraphrased)
General VR use in health care (n=185, 17.52%)	113	Tweets that provided information about the use of VR in health care broadly (ie, not directly linked to use in a particular discipline or condition). This included links to or promotion of online events such as webinars and conferences about the use of VR in health care (n=92); a link to an article or blog that provided an overview of VR use and opportunities in health care (n=56); information and news updates about the VR health care market including trends, predictions, and companies to watch (n=37).	Only 2 more days to sign up for our online event about #VR #tech in the health industry [URL] #Healthcare #VirtualReality #future<br><br>The #VR health care market is predicted to grow in the future [URL] #Tech #Health #Virtual
Pain and distraction (n=108, 10.23%)	84	Tweets that were related to using VR for pain management or distraction from pain or medical procedures (n=95) (eg, pre-operative anxiety; management of chronic back pain) and in end-of-life care (n=13) (eg, to reduce feelings of pain, isolation, and anxiety).	NEW article #VR to reduce #pain #backpain [URL]<br><br>See how #VirtualReality can help those receiving palliative care [URL]
Brain injury rehabilitation (n=45, 4.26%)	43	Tweets about the use of related to acquired brain injuries (eg, traumatic brain injury, stroke), concussion, and VR neurorehabilitation companies and products.	#Research shows that #VR #therapy can help in #stroke rehabilitation. #neurology [URL]
Neurodegenerative conditions (n=30, 2.84%)	22	Tweets about the use of VR for people with Parkinson's disease (eg, eye tracking, gait training), dementia (eg, reducing agitation), multiple sclerosis (eg, upper limb retraining), or VR education resources about neurodegenerative conditions (eg, embodiment VR platforms).	Check out our #VR module on #dementia [URL]<br><br>Research shows that #VR can help those living with #Parkinson's [URL]

Coding Category (Number of Original Tweets, %)	Number of @Users	Category Description	Example Tweet (Paraphrased)
General rehabilitation (n=29, 2.75%)	26	Tweets that provided information about VR use in a range of rehabilitation settings (eg, physical therapy, vestibular rehabilitation, amputees, balance and gait, spinal injuries, non-specified rehabilitation).	Awesome paper on #VR at home #gait training for #rehab. Could be important with #COVID #online #VR #tech
3D modeling and medical image viewing (n=26, 2.46%)	21	Tweets that provided information or news related to the use of VR for 3D modeling in health care. Examples included using VR for viewing medical images, drug design, viewing virus structures, designing medical models. Medical animation VR platforms were also promoted.	Medical scans #CT are becoming immersive with #VR #AR #virtualreality #HealthTech [URL]
Visual impairments (n=24, 2.27%)	24	Tweets about the use of VR for visual impairments. Uses included at home glaucoma care via remote monitoring, treating vision impairments, assisting sight of people with low vision, eye tracking assessments.	New #technology using #VR can help people with vision problems see more clearly [URL]
Senior care (n=23, 2.18%)	21	Tweets about using VR in skilled nursing facilities and for seniors to maintain connections, reduce loneliness and social isolation by connecting with others, and to reduce anxiety (especially during the COVID-19 pandemic).	Our reporter explores the use of #VR in #nursing facilities [URL] #news #health #seniorcare
Other (n=66, 6.25%)	47	Disciplines or conditions that were tweeted about less frequently:<br>- Patient and public education regarding health conditions, medical procedures, and disciplines (n=16)<br>- Paediatric care, including for cerebral palsy and social care (n=12)<br>- Autism – included research articles, videos, and webinars (n=11)	Great interview about using #VirtualReality for #social skills for #autism [URL]<br><br>The potential benefits of #VR in health care for better hospital and facility #design are awesome. Read more [URL]<br><br>NEW STUDY: The effects of #VR on #physical

Coding Category (Number of Original Tweets, %)	Number of @Users	Category Description	Example Tweet (Paraphrased)
		- Exercise – use of VR for promoting exercise and physical activity to improve health (n=8)<br>- Role in telehealth and remote research, especially during the COVID-19 pandemic (n=6)<br>- Auditory training and performance (n=4)<br>- Hospital and health facility design (n=3)<br>- Practicing disclosing sensitive health information to others (n=3)<br>- Empathy building (n=2)<br>- Pharmacy (n=1)	activity [URL]<br><br>#VR can be used to teach safe behaviours and educate patients about their health [URL] #Tech #VR #AR #health #education

This is a Multimedia Appendix to a full manuscript. For full copyright and citation information see doi: 10.2196/45168
