# Supplementary material for: Exploring Discussions About Virtual Reality on Twitter to Inform Brain Injury Rehabilitation: Content and Network Analysis
Source: J Med Internet Res. 2024 Jan 19;26:e45168. doi: 10.2196/45168 (PMC10837760; doi:10.2196/45168)
Supplement: Multimedia Appendix 4 [file jmir_v26i1e45168_app4.pdf]

Details on Original Tweets Related to 'Talking About VR' (N=3506)

Sub-Category (Number of Original Tweets)	Number of @Users	Description	Example Tweet (Paraphrased)
**General conversations about VR (n = 2904, 82.83%)**			
VR experiences (n=1229)	867	@Users provided details, descriptions, and opinions about a direct experience with VR (past or future). @Users also shared their VR purchases (eg, HMDs, accessories, games), or commented on their attendance at past or future VR events. Many of these tweets contained videos or photos of @users using VR, their VR set-up, or screencaps of their VR experiences.	It has arrived!!! Finally got my HMD #VR #oculus<br><br>Had an incredible time in #VR<br><br>Had a blast at our meetup in #VR. Was great to listen to all on the panel
Commentary on VR news or content (n=554)	374	Tweets where @users commented on or shared opinions about VR that was not related to a direct experience they had with VR (eg, commenting on news about VR apps, hardware, content updates, other @users' VR experience, VR companies).	Can't wait for this #VR game! Looks amazing #fun #VirtualReality<br><br>Not sure about the new update for [VR game]. Guess we will have to give it a go #gamer #VR
General commentary and conversations (n=520)	371	Tweets were @users discussed a range of uses, topics, and opinions related to VR. Most of these were conversational tweets directed at other @users.	[@user] I'm glad that you're enjoying the #VR game<br><br>[@user] I would say that this #VR headset delivers a good gaming experience, but I can't wait for the next release

Sub-Category (Number of Original Tweets)	Number of @Users	Description	Example Tweet (Paraphrased)
VR potential and ideas (n=295)	266	Tweets were @users expressed their opinion and discussed the potential of VR both generally and in specific industries. This included the potential of VR in industries and uptake due to the COVID-19 pandemic (eg, for remote work, education, events). Alternatively, some @users discussed barriers to VR and its general potential. @Users suggested ideas for VR games, or expressed wanting cross platform games or support to be developed.	I see social #VR apps as a way to integrate custom worlds. What's stopping us? #VirtualReality #future #tech<br><br>I have a suggestion. How cool would [video game] be in #VR? Someone make it happen!
Development insights and content (n=220)	162	@Users described VR prototypes or content that they were developing and shared related pictures or videos.	Still fixing up the shading but here's a sneak peek at my #VR development #GameDev
VR challenges (n=86)	67	Tweets where @users expressed opinions or discussed challenges with VR and the VR industry – including cost, privacy, lack of access, quality, and interaction mechanisms. Did not include tweets that provided information about personal experiences with VR issues or where @users sought advice or opinions.	I think lack of hardware is an issue for many #VR developers<br><br>#VR isn't there in terms of quality of avatars and interaction methods. I feel more connected in online games.
**Seeking advice or opinions about VR (n=439, 12.52%)**			
VR hardware and software (n=147)	123	@Users asked for advice or opinions related to VR games (eg, which games/apps to play, stream or purchase, game features), or questions or wanted opinions about HMDs (eg, which HMD to purchase, are HMDs worth purchasing, cost of HMDs).	What would be a fun #VR game to play? #subscribe #games #VirtualReality #vr<br><br>Looking to buy #VR, what is the best #HMD? #oculus #HTC

Sub-Category (Number of Original Tweets)	Number of @Users	Description	Example Tweet (Paraphrased)
VR features, design, and development (n=103)	71	@Users asked for opinions or advice related to features of VR and development (eg, game engines, graphics card requirements, streaming, technical barriers).	What mode are you using for #VR scenes? #GameDevs #unity #indiedev
Issues with VR (n=63)	23	@Users sought advice about issues that they had experienced with VR (eg, broken hardware, graphics glitches, connectivity problems).	Been trying to fix black screen #fail with my VR, any ideas you think can help?? #VirtualReality
VR industry (n=50)	45	@Users asked for opinions or connections related to the VR industry.	Who are some #VR creators we can follow? #GameDev #technology
VR uses (n=24)	19	Seeking opinions about VR experiences or potential uses.	Anyone think #VR concerts will take off?
Other (n=52)	48	Tweets that did not form larger content categories.	Do you like watching 24 hours in #VR videos?
**User preferences and feedback about VR experiences (n=163, 4.65%)**			
Side effects of VR use (n=55)	47	Tweets with comments or information about VR side effects (eg, headaches, nausea, discomfort), including direct experiences and information about reducing potential for side effects.	Ughh I love playing #VR but this game has got me feeling so sick<br><br>I recommend taking it slow with seated #VR games first
Issues with VR (n=50)	44	@Users shared and described problems they were experiencing with VR including with hardware (eg, HMDs, hand controllers), apps, audio issues, graphics issues, error messages.	Can't get my VR game to work properly with audio #fail #VR #gamer
Design preferences or opinions (n=32)	31	@Users described or discussed preferences or opinions about design aspects of VR software or hardware (eg, field of view, degrees of freedom, scaling, image resolution).	I prefer third person #VR games over first person view #games #gamer #VirtualReality

Sub-Category (Number of Original Tweets)	Number of @Users	Description	Example Tweet (Paraphrased)
Accessibility (n=26)	20	Tweets related to making VR accessible for people who may experience limitations with access (eg, visual or physical impairments).	#Accessibility in #VR matters and developers should make experiences with custom options and seated games

This is a Multimedia Appendix to a full manuscript. For full copyright and citation information see doi: 10.2196/45168
